# Dental Instruction in the Public Schools

**Published:** 1903-05-15

**Authors:** Delos Fall


THE
DENTAL REGISTER.

Vol. LVII. May 15, 1903. No. 5.

### DENTAL INSTRUCTION IN THE PUBLIC SCHOOLS.

BY PROF. DELOS FALL.

Address before the Southwestern Michigan Dental Association, April 8, 1903.

I am not certain of the propriety of assuming to direct your attention to-day to a subject which you perhaps understand, or appreciate the significance of its discussion in this meeting better than I do. But I trust you may not think there exists any great difference at least in the desire on the part of your profession and mine to earnestly engage in any matter that shall make for better health of the children in our public schools.

I am not sure but that one of the evils of our time is that too much energy and time is wasted in individual efforts. We are very apt to sit still and watch some one who has a faculty for, or a desire to do some good benevolent work, rather than to take our places along with him and lend our influence if not our cordial co-operation. The need of to-day is for more team work. It is my business to superintend the work of secondary education in this State and am planning in every way to enlist the co-operation of every person, or class of persons, who may have an interest in this work with me, so that the best possible educational methods and machinery may be placed at the disposal of the children of to-day, who are to do the world's work to-morrow. I think I have made a valuable discovery in the department which you follow as your daily occupation, and this has brought me here to present my view of this situation with the hope of engaging your thought and broadening my own outlook.

Ten years ago, in studying the subject of hygiene as it was then taught in our secondary schools, I became convinced that much unnecessary work and not a little of erroneous teaching was being done, and that the entire subject needed remodeling. The text-books in use contained much anatomy, a little physiology and a very little practical hygiene. The teachers themselves were not properly informed as to sanitary principles, and naturally taught the subject most imperfectly if not in most cases falsely.

To meet the conditions I then found, I prepared a syllabus to be used as a manual for preparing teachers to do this work in a satisfactory manner. I confess now that this work was faulty, as it left out one of the most important principles of sanitary instructions—that of the nature of communicable diseases. There was nothing in it as to the nature of these diseases, the manner in which they are disseminated, and consequently no intelligent instruction as to the methods employed to prevent their spreading because of unsanitary conditions.

At that time but one or two colleges in the country taught sanitary science scientifically. In 1886, I became a member of the State Board of Health and endeavored to have this organization take some practical means to disseminate the principles of sanitary science among the people, so as to do what might be to conserve the public health, and one of my associates on the Board thought because I was not a physician I had no qualifications for deciding what means should be used to warn the people of their dangers, or to suggest preventive measures. But I maintained that as an officer whose duty it was to conserve the public health, and a teacher by profession, it became a matter of duty that I utilize all my knowledge in accordance with my best judgment to educate through the public schools the children in matters of general hygiene. I am pleased to find that there is now a strong sentiment in favor of my position and that the dental profession is also interested in doing what it can to preserve the public health, by instructing school children in the care of the teeth. In 1895, our Legislature passed a law making it obligatory that public-school teachers should impart the general principles of communicable diseases, and in some States these laws are rigidly enforced.

The State Board of Health takes special pains to put into the hands of instructors in the schools the latest and best knowledge of the subjects of general hygiene, especially in regard to epidemic of communicable diseases. Local Boards of Health are also actively interested in enlightening their communities as to the nature of such diseases and the best means for suppressing or preventing their propagation. It would be easy to take the statistics of our State Health Officer and prove that epidemics are now rare and that many lives are saved annually because of the enlightenment of the people on sanitary measures through the educational efforts of the schools and health boards.

One of the most important measures which has been worked out in recent years is that of gaining the co-operation of the medical profession, in visiting the schools at proper intervals for the purpose of discovering the presence of children having incipient symptoms of diseases of an infectious nature.

It has long been known, that the public school is always a congested center of infection, a focus toward which those contagious diseases incident to child-life—diphtheria, scarlet fever, measles, whooping-cough and the like—naturally and inevitably make their way from infected homes, and from which those same diseases travel outward to other homes. A study of the vital statistics conclusively shows that coincident with the beginning of school in September of every year there is a maximum of sickness and death from scarlet fever and diphtheria, and that a minimum prevalence is observed during the vacation months. For example, scarlet fever may and quite frequently does occur in a home without the notice or attendance of a physician, and without coming under the observation of the health officer, and pupils are sent to school before the period of desquamation has passed. Here, by association of the pupils of the same grade, they become new centers of infection and many cases may arise from the carelessness and ignorance of the one case. The time has come when there should be for all the schools of the cities and villages such daily medical inspection as will anticipate and check outbreaks of disease which inevitably will occur without such inspection and supervision.

Work of this nature has been carried on for a number of years in Europe, notably in the city of Brussels, where daily medical inspection has been practiced with great success. The city of Boston in 1894, at a time when an epidemic of diphtheria showed the authorities that something of a radical nature must be done, entered upon a system of daily medical inspection of the schools. Later the health authorities of New York inaugurated a similar work.

Boston has a most carefully-planned system of daily inspection of the schools of that city. The last published report includes the work of fourteen months. During that time very many cases of contagious diseases had been found in the schools, which required early diagnosis and removal from the school, and also many cases of sickness which were referred to the family physician for decision as to what should be done. By a preliminary examination each morning, conducted by the teacher in charge, such pupils were selected out as evidenced any sign that they were sick or likely to be sick from a communicable disease, and sent to the superintendent's office or other room provided for the purpose. Here they were met by the Medical Inspector, each case carefully examined by him, and each pupil disposed of according to the judgment which he formed. Of 16,790 pupils examined, 10,737 cases of illness were found, during the fourteen months, and of these 77 were cases of diphtheria, 28 of scarlatina, 116 of measles, 28 of chicken-pox, 69 of pediculosis, 47 of mumps, 47 of scabies, 33 of whooping-cough. There were also a large number of contagious diseases of the eye, and over two thousand cases of follicular tonsilitis, which we know is an infectious disease.

The late reports from the New York Bureau, whose work is laid out on plans similar to those obtaining in Boston, are very interesting. The inspection report of the first four day's work of this Board shows that over four hundred children were suffering from contagious diseases, of which 10 were diagnosed as diphtheria, 2 of measles, 1 of scarlet fever and 6 of mumps. The report for the week ending April 10th shows that 364 children were excluded. The cases were divided as follows: Measles, 2; diphtheria, 13; scarlet fever, 1; croup, 3; whooping-cough, 4; mumps, 10; contagious eye diseases, 59; parasitic diseases, 227; chicken-pox, 15; skin diseases, 19. That such results have been obtained is not to be wondered at, when we recall the indifference and carelessness and ignorance of parents and others who would be supposed to have at heart the welfare of the little children. Of the extent of the evils which were thus prevented, we can only guess; they can never be known except approximately.

Here are facts which, at least, should open your eyes to the possibilty of what may happen in our schools at any time. Dipththeria, with all its terrors, is not confined to Boston or New York. It too frequently and too suddenly occurs in many of the schools of Michigan, and the question arises whether it is not only right, but the duty of the school authortiies to see that the daily assembling of pupils be had without physical or moral harm; to know, when pupils are gathered together in the rooms in which they are to spend many hours, that these rooms shall not only be properly warmed, lighted and ventilated, but that contagious diseases are not only not present, but not possible. To secure this condition of affairs requires expert service such as the schools at present, in the persons of the superintendent and teachers, can not give. Expert knowledge concerning the imminence of communicable diseases is only possessed by the trained medical inspector, and it is only through his alliance with the school authorities that we may hope to give the schools that prevision and supervision necessary to prevent the outbreak of the contagious scourges of young life. There is no doubt but that in the significant sanitary educational work which is now going on in our Michigan schools there results an ever increasing knowledge of diseases, their causes and their modes of spread, as well as knowledge by which their spread may be restricted and prevented, and that our teachers are training themselves and thereby coming into that degree of effectiveness in sanitary matters by which it will be possible to prevent much of human suffering; and yet their eyes are not fully opened so that they quickly see the evidences of the approach of disease as can the trained physician whose vision has been sharpened by expert knowledge of those evidences.

It seems to me that we have overlooked one of the most important places of inspection, and that is the mouth. The influence of a diseased mouth in spreading disease should be apparent to every one when we think of the chances occurring from the exhalation through the mouth of badly contaminated air, the spittle, drinking cup and other opportunities of this sort. And it occurs to me that a dental as well as a medical inspection should go hand in hand that we may secure the complete evidence and apply proper preventive measures.

The great difficulty in making practical such suggestions is the fact that no money is set apart by our school boards to recompense medical or dental practitioners for services which are necessarily exacting and which require pecuniary compensation, if they are to be done at all adequately. The only present hope is that the professional men may be loyal to their traditions and make a sacrifice of time and money in behalf of a worthy and benevolent object, until such time as their services have demonstrated the necessity for them, and then we may expect a reasonable compensation.

I have no doubt that the dentist who is continually looking at the mouth will be able to detect there the symptoms of encroaching disease which would be wholly overlooked by the medical examiner. I need not tell you that the mouth is a veritable hot-bed for the propagation of microorganisms and some of these the most virulent and destructive. I thank you for the opportunity of presenting this subject and trust you will give it your most earnest consideration.

### DISCUSSION.

DR. J. TAFT: In the past, the function of the physician, as we look at it, was to treat diseases, and there are possibly some physicians who still take this view of their mission. But this is not the benevolent nor the professional view of the cultured physician of to-day. He strives not only to cure disease when he finds it, but is equally concerned as to its prevention. The dental profession has always taken a high stand in the matter of prophylaxis, and the dentist's operations have been made with the purpose of not only restoring lost dental organs or structures, but to prevent recurrence of conditions which threaten the welfare of the teeth and their associated structures, so that the first step in the important act of digestion should be performed with the greatest possible perfection. In the matter of the part which the mouth takes in the spread of contagious diseases, our profession is as well qualified to speak as the medical scientist, Some of the best researches in the field of bacteriology have been made by a member of our profession, Dr. Miller, an American dentist practicing in Berlin, Germany, who found and studied over thirty different micro-organisms in the human mouth, and his work has a well-credited place in the classics of this important if not fundamental branch of sanitary science.

As to the efforts the speaker has made or recommended, it seems to me that if the health officers of every city and county in every State would attend strictly to the business which properly belongs to them, there could be no such thing as an epidemic of disease. But the difficulty seems to be that these officers are not properly selected, or are restricted in their opportunities so universally that epidemics are all too common, and will so continue until the people become sufficiently intelligent on this matter to be willing to elect qualified men to this important office and clothe them with proper powers and sufficient salary to make their work what it can and should be. The future looks hopeful, legislation is becoming more intelligent everywhere, and the general public is being enlightened.

I don't know that the dentist can, in any public way, make his work felt, but he can do whatever comes into his care as a practitioner in such a skillful manner as to ward off disease and by systematically instructing his patients in the ordinary toilet applications he will be doing much to prevent the possibilities of spreading disease from the mouth. By keeping the teeth in as perfect a condition as is practicable, he will aid in the general health by enabling the patient to thoroughly masticate the food for proper digestion.

Dentists can also, by co-operating with the physician, do much to detect the encroachment of insidious diseases which manifest their presence in the mouth where the physician would overlook them, because he does not give as careful attention to the oral structures as the dentist. Dentists should be able to detect aberrations from the normal in the mucous membrane and if there is no local cause which may account for them he should refer such patients to the family physicians for more experienced examination. In my opinion, the teeth of children receive far too little attention from the dentist. The mouths of children in school ought to be examined carefully at least every month, and in some cases more frequent examinations should be made. It is not practicable for dentists to undertake technical work in the school room, but regular and frequent examinations and reports sent to the parents would do much to prevent the premature loss or serious diseases of the teeth. There are other conditions closely associated with the teeth that need looking after in a public way. The habit of mouth breathing is all too common and is invariably due to interference with the respiratory passages of the nose, which could be easily remedied by slight operations. These conditions not only interfere with the intellectual progress of the children and so hinders the members of the class by taking an undue amount of the teacher's time, but they are prolific sources of infections which threaten the health of all the children associated with such an afflicted pupil.

